# Supplementary material for: Development of an intervention to facilitate dissemination of community-based training to respond to out-of-hospital cardiac arrest: FirstCPR
Source: PLoS One. 2022 Aug 24;17(8):e0273028. doi: 10.1371/journal.pone.0273028 (PMC9401178; doi:10.1371/journal.pone.0273028)
Supplement: S1 File — (DOCX) [file pone.0273028.s001.docx]

**SUPPLEMENTARY INFORMATION S1**

**Supplement S1: LIST OF STAKEHOLDER ORGANISATIONS**

| **Organisation name** | **Website and more information** | **Type** |
| --- | --- | --- |
| NSW Ministry of Health | <https://www.health.nsw.gov.au/about/ministry/Pages/default.aspx> | Government |
| NSW Ambulance | <https://www.ambulance.nsw.gov.au/> | Government |
| Western Sydney Local Health District | <https://www.wslhd.health.nsw.gov.au/> | Government |
| NSW Data Analytics Centre | <https://data.nsw.gov.au/nsw-data-analytics-centre> | Government |
| City of Parramatta Council | <https://www.cityofparramatta.nsw.gov.au/> | Government |
| Surf Life Saving NSW | <https://www.surflifesaving.com.au/community-programs> | Education and training service provider |
| Michael Hughes foundation | [https://www.mhf.life](https://www.mhf.life/) | Education and training service provider |
| The National Heart Foundation of Australia | <https://www.heartfoundation.org.au> | Community organisation |
| Take Heart Australia | <https://www.takeheartaustralia.org> | Community organisation |
| Heart Support Australia | <https://www.heartsupport.org.au/> | Community organisation |
| Westmead Applied Research Centre, The University of Sydney | <https://www.sydney.edu.au/medicine-health/our-research/research-centres/westmead-applied-research-centre.html> | Research institute |
