# Supplementary material for: Development of an intervention to facilitate dissemination of community-based training to respond to out-of-hospital cardiac arrest: FirstCPR
Source: PLoS One. 2022 Aug 24;17(8):e0273028. doi: 10.1371/journal.pone.0273028 (PMC9401178; doi:10.1371/journal.pone.0273028)
Supplement: S2 File — (DOCX) [file pone.0273028.s002.docx]

**SUPPLEMENTARY INFORMATION S2**

**Supplement S2**. **Screening for selection of publicly-available material** (videos and factsheets)

**VIDEOS**

- Video Origin: (country)
- Video Creator (if accredited organisation)
- Video length (time in minutes and seconds)
- Topics covered (e.g., Demonstration of Hands-only CPR, rescue breaths, or AED use)
- Clinically correct demonstration of CPR or AED use (Y/N)
- Mention of the local 000 emergency number (Y/N)
- Appropriate language for lay persons (Y/N)
- Relevant and coherent video segments and/or images (Y/N)
- Quality images (Y/N)
- Clarity of actors’ voices/voiceover (Y/N)

**FACTSHEETS**

- Factsheet creator (if accredited organisation)
- Topics covered (e.g., Hands-only CPR, rescue breaths, or AED use)
- Local 000 emergency number listed (Y/N)
- Content clinically correct (Y/N)
- Engaging (Y/N)
- Appropriate language for lay persons (target audience lay or professional)
- Relevant and clear images (Y/N)
- Relevant and concise text (Y/N)
- Appropriate text density (Y/N)
