# Supplementary material for: Development of an intervention to facilitate dissemination of community-based training to respond to out-of-hospital cardiac arrest: FirstCPR
Source: PLoS One. 2022 Aug 24;17(8):e0273028. doi: 10.1371/journal.pone.0273028 (PMC9401178; doi:10.1371/journal.pone.0273028)
Supplement: S3 File — (DOCX) [file pone.0273028.s003.docx]

**SUPPLEMENTARY INFORMATION S3**

**Supplement S3: Sample of messages and material in final digital package**

**Examples of message sentence and accompanying intervention material***

| **Message** | **Accompanying content (video/factsheet)** | **Material creator / owner** |
| --- | --- | --- |
| Hello [participant_name]  ‘Most cardiac arrests occur at home. CPR can double the chance of survival. Please click the link below to watch a brief video explaining how to respond to a cardiac arrest in the community.’ | Brief but comprehensive video demonstrating the key steps involved in responding to a cardiac arrest- focus on Call, Push, Shock. | Created by: Ambulance Victoria, Australia [1,2] |
| Hello from FirstCPR  Take note of where defibrillators (AED) are located in your community, [participant_name]. Look for AED signs at the shops, clubs, and other public places. Click the link to images of possible locations.’ | A factsheet showing images of where an AED could be located (e.g., shopping centre) and what the sign looks like. | Created by: The FirstCPR study team |
| Bystander CPR can save lives. Please click on the link below to watch a brief video of a survival story. | Link to a brief video that highlights a survivor story and emphasises the important role played by the witness/bystander. | Created by: Ambulance Victoria [2] |
| Hello [participant_name], calling 000 from the Emergency+ app automatically provides your location to NSW ambulance - saving precious time. Download this free app by clicking the link. | Link to the ‘Emergency+ app’ factsheet information that enables the download of this free app from commonly used and secure platforms.  The app uses GPS functionality built into smart phones to help a Triple Zero (000) caller provide critical location details required to mobilise emergency services. | App publicly available and provided by Australian Emergency services [5] |
| Hello from FirstCPR,  Only 1 in 10 survive an out-of-hospital cardiac arrest. Greg Page, the famous Yellow Wiggle shares his story. Please click on the link below to watch his short video. | Link to a brief video narrated by a famous personality on how he survived a cardiac arrest and again highlighting the role of the bystander. | Created by Surf Life Saving NSW [1, 4] |
| Automated External Defibrillators (AED) are safe to use, [participant_name]. And you can save a life using one in a cardiac arrest. Click the link to view a poster highlighting key facts about AEDs. | A factsheet that addresses the key barriers about a using a defibrillator. | Created by NSW Ministry of Health [3] |
| ‘Hello from FirstCPR,  Remember - you can do effective CPR even without the mouth-to-mouth breaths - called hands-only CPR and demonstrated here’. | A short, animated video that emphasises Call Push Shock thus demonstrating hands-only CPR. | Created by: Australian Capital Territory Ambulance [4]. |

*Note: All materials produced or created by a third party were obtained with appropriate permissions for use in the study

**References for access to sample digital material described above**

1. Youtube channels available at [www.Youtube.com](http://www.Youtube.com) accessed Jan 2020 to Dec 2021.

2. Ambulance Victoria. (n.d.) Home (Facebook page). Facebook. Retrieved July 2020

3. NSW health. *Cardiac arrest information for consumers and organisations.* Available at <https://www.Health.Nsw.Gov.Au/cardiacarrest/pages/default.Aspx> accessed July 2021.

4. Vimeo videos available at [www.vimeo.com](http://www.vimeo.com) accessed Jan 2021 to Dec 2021

5. App Store to access Emergency Plus app available at <https://apps.apple.com> and <https://play.google.com> accessed from June 2021 to Dec 2021
