# Supplementary material for: Development of an intervention to facilitate dissemination of community-based training to respond to out-of-hospital cardiac arrest: FirstCPR
Source: PLoS One. 2022 Aug 24;17(8):e0273028. doi: 10.1371/journal.pone.0273028 (PMC9401178; doi:10.1371/journal.pone.0273028)
Supplement: Attachment — Submitted filename: CPR review.docx [file pone.0273028.s004.docx]

Strengths:

This project meets a pressing community and public health need. The manuscript is written in clear language. This work is well positioned in the context of other outreach efforts. The theory-guided and collaborative approach involving multiple stakeholders is well reasoned and admirable. The key messages, encouraging hands-only CPR, Call, Push, Shock, etc. are on target.

Limitations:

One limitation is that this study does not assess the impact of the outreach program, which presumably will happen in the future.

The major criticism of the paper regards generalizability. This project appears to meet a local need in NSW Australia. What should a reader in the US or Europe or elsewhere take away from this? Why should this work matter to a global audience? Readers have no way of knowing whether the tool that was developed will work even in the location it is designed for, even less about how it might be useful more broadly.

Relating to that point, the authors mention that messaging needed to be matched to the specific audience and that resources with British and American accents and regional language such as “being sued” that might impair the impact of that messaging. Do you suggest that other places should similarly aim for messages that resonate with that specific area, using regional terms, referring to locally relevant shared cultural references, and avoiding accents?

Additional points:

Line 345. regarding “minimal adaptations”, do you mean in when generalized to other places in Australia? What about other areas? Are you suggesting that the tool you are developed will be broadly useful? or that the technique that you employed to bring the tool about will be broadly useful? Is specific tailoring a lesson that can be applied when outreach efforts are pursued elsewhere?

p 396. Was the survey group non-random in a way that could bias the results? On p 381. you write “Our approach also aims to increase the availability of education and training within communities that are often left out of such interventions.” Was the survey group (n=64) representative of the target audience and its diversity?

P 15. Avoid redundancy: There’s no point in providing a summary if it is almost exactly repeated in the response in quotes. Overall, Table 1 is okay, but I am not convinced of the need to include individual quotations unless there was a theme that emerged or if the response resonated with the investigators for some reason that should also be explained to the reader. It might be overstating a result to mention a survey response in the text (.e.g about jokes/comedy, line 275) and also in a summary statement followed by a respondent quotation saying the same thing. Was this one thing that one person said, or was it a theme that came up repeatedly?

P19. line 318. “slides”? I know what you mean of course, but younger I know what you mean, of course, readers might not. How about visual resource, or something like that?
