# Supplementary material for: Development of an intervention to facilitate dissemination of community-based training to respond to out-of-hospital cardiac arrest: FirstCPR
Source: PLoS One. 2022 Aug 24;17(8):e0273028. doi: 10.1371/journal.pone.0273028 (PMC9401178; doi:10.1371/journal.pone.0273028)
Supplement: Attachment — Submitted filename: Response to Reviewers.docx [file pone.0273028.s005.docx]

We would like to thank PLOS ONE editors and reviewers for the time spent reviewing our manuscript and for the opportunity to respond to these useful comments. Responding to this feedback has strengthened the manuscript, as detailed below and via tracked changes in the manuscript.

**Response to Reviewers’ comments**

**Strengths:**

**This project meets a pressing community and public health need. The manuscript is written in clear language. This work is well positioned in the context of other outreach efforts. The theory-guided and collaborative approach involving multiple stakeholders is well reasoned and admirable. The key messages, encouraging hands-only CPR, Call, Push, Shock, etc. are on target.**

**Limitations:**

**One limitation is that this study does not assess the impact of the outreach program, which presumably will happen in the future.**

Yes, this is correct. This is a description of the intervention and its development. The evaluation and impact will be reported separately.

**The major criticism of the paper regards generalizability. This project appears to meet a local need in NSW Australia. What should a reader in the US or Europe or elsewhere take away from this? Why should this work matter to a global audience? Readers have no way of knowing whether the tool that was developed will work even in the location it is designed for, even less about how it might be useful more broadly.**

The takeaway message for a global audience should be that broad-brush programs may not be applicable in all contexts and settings and there is a need for culturally and locally appropriate programs that maximize collaborations and integrate locally produced materials. The overall process could provide a template for other international groups to design similar programs and localise them using materials specific to their culture, language, and accent. We have revised the manuscript to reflect this message in the following sections:

pg 2, lines 34-35;

pg 3, lines 52 and 58;

pg 5, lines 88-90 and 99-100;

pg 6, line 101, 111;

pg 8, line 159;

pg 12, lines 230-234;

pg 22, lines 331, 345-347

pg 24, lines 381-387

**Relating to that point, the authors mention that messaging needed to be matched to the specific audience and that resources with British and American accents and regional language such as “being sued” that might impair the impact of that messaging. Do you suggest that other places should similarly aim for messages that resonate with that specific area, using regional terms, referring to locally relevant shared cultural references, and avoiding accents?**

As described above, we believe it is important to localise materials for the target audience. As described in our paper, this includes incorporating shared cultural references, familiar accents and imagery that reflects local communities, so that it resonates as culturally familiar and appropriate. See amendments on pg 24 lines 381-387

**Additional points:**

**Line 345. regarding “minimal adaptations”, do you mean in when generalized to other places in Australia? What about other areas? Are you suggesting that the tool you are developed will be broadly useful? or that the technique that you employed to bring the tool about will be broadly useful? Is specific tailoring a lesson that can be applied when outreach efforts are pursued elsewhere?**

Yes, one of the key messages is that specific tailoring to the local context should be an important consideration along with seeking input from key stakeholders and using locally produced/created material. We have clarified this on pg 22-23, lines 378-384.

**p 396. Was the survey group non-random in a way that could bias the results? On p 381. you write “Our approach also aims to increase the availability of education and training within communities that are often left out of such interventions.” Was the survey group (n=64) representative of the target audience and its diversity?**

While the respondents were a non-random sample, we did maximise diversity of respondents from different age groups and socio-demographic backgrounds, including professionals in health and research as well as those from non-health occupations. We acknowledge that a representative sampling approach would be less biased and have noted this as a limitation:

pg 27, line 439, 447, and 448-453.

**P 15. Avoid redundancy: There’s no point in providing a summary if it is almost exactly repeated in the response in quotes. Overall, Table 1 is okay, but I am not convinced of the need to include individual quotations unless there was a theme that emerged or if the response resonated with the investigators for some reason that should also be explained to the reader. It might be overstating a result to mention a survey response in the text (.e.g about jokes/comedy, line 275) and also in a summary statement followed by a respondent quotation saying the same thing. Was this one thing that one person said, or was it a theme that came up repeatedly?**

We have now removed quotes in the updated Table 1 (pg 16) to just provide a summary of the preferences for material based on review and feedback from participants.

**P19. line 318. “slides”? I know what you mean of course, but younger I know what you mean, of course, readers might not. How about visual resource, or something like that?**

We agree with this point and have now reworded in several places:

pg 10, lines 189-190;

pg 11, line 211;

pg 22, lines 338-339; and

pg 23, line 352-355.

**RESPONSE TO EDITORS’ COMMENTS**

**Journal Requirements:**

**When submitting your revision, we need you to address these additional requirements.**

**1. Please ensure that your manuscript meets PLOS ONE's style requirements, including those for file naming. The PLOS ONE style templates can be found at** 
[**https://journals.plos.org/plosone/s/file?id=wjVg/PLOSOne_formatting_sample_main_body.pdf**](https://protect-au.mimecast.com/s/VhyzC0YKPvi2QADNGFWzQ58?domain=journals.plos.org) **and** 
[**https://journals.plos.org/plosone/s/file?id=ba62/PLOSOne_formatting_sample_title_authors_affiliations.pdf**](https://protect-au.mimecast.com/s/RNaICgZ0N1ilKzxEAfE7a8m?domain=journals.plos.org)

We have aligned the manuscript to meet PLOS ONE’s formatting and style requirements.

**2. Please provide additional details regarding participant consent. In the ethics statement in the Methods and online submission information, please ensure that you have specified (1) whether consent was informed and (2) what type you obtained (for instance, written or verbal, and if verbal, how it was documented and witnessed). If your study included minors, state whether you obtained consent from parents or guardians. If the need for consent was waived by the ethics committee, please include this information.**

Phase two of this study involved sending out a link with shortlisted intervention material for participants to review, comment, and provide anonymous feedback. The link was sent via e-mail and consent was ‘implied’ if participants proceeded to click the link to provide anonymous feedback or they could choose to opt out by not proceeding to click the link. We have updated the manuscript on pg 10, lines 195-197 to reflect this implied consent.

**3. We noted in your submission details that a portion of your manuscript may have been presented or published elsewhere.**

Yes, a paragraph that summarizes a section of the results was included in a protocol paper for the FirstCPR study. A copy of the related work has been uploaded as a 'Related Manuscript file'.

**Please clarify whether this [conference proceeding or publication] was peer-reviewed and formally published. If this work was previously peer-reviewed and published, in the cover letter please provide the reason that this work does not constitute dual publication and should be included in the current manuscript.**

Yes, confirming that the related publication was peer-reviewed, formally published, and can be found at doi:10.1136/bmjopen-2021-057175 and cited as *Munot S, Redfern J, Bray JE, et al. Improving community-based first response to out of hospital cardiac arrest (FirstCPR): protocol for a cluster randomised controlled trial. BMJ Open 2022;12:e057175.*

This publication describes the detailed protocol of the FirstCPR cluster randomised trial. Although it mentions the FirstCPR intervention, it does not detail the process of developing the intervention package, which is what the current manuscript aims to describe. We believe there is merit to describing this formative process in detail to provide generalizable knowledge on building a robust and scalable intervention package that can serve as a template for the development of similar large-scale public health and educational interventions around the globe. It was not possible to detail this process in the FirstCPR protocol publication and therefore merits a separate publication.

**4. We note that Supplementary information S3 includes an image of a [patient / participant / in the study]. 
As per the PLOS ONE policy (**[**http://journals.plos.org/plosone/s/submission-guidelines#loc-human-subjects-research**](https://protect-au.mimecast.com/s/rg6ECjZ1N7ijDxMonTnj3OE?domain=journals.plos.org)**) on papers that include identifying, or potentially identifying, information, the individual(s) or parent(s)/guardian(s) must be informed of the terms of the PLOS open-access (CC-BY) license and provide specific permission for publication of these details under the terms of this license. Please download the Consent Form for Publication in a PLOS Journal (**[**http://journals.plos.org/plosone/s/file?id=8ce6/plos-consent-form-english.pdf**](https://protect-au.mimecast.com/s/bIWFCk81N9tnAJ62OukjQPt?domain=journals.plos.org)**). The signed consent form should not be submitted with the manuscript, but should be securely filed in the individual's case notes. Please amend the methods section and ethics statement of the manuscript to explicitly state that the patient/participant has provided consent for publication: “The individual in this manuscript has given written informed consent (as outlined in PLOS consent form) to publish these case details”.**

**If you are unable to obtain consent from the subject of the photograph, you will need to remove the figure and any other textual identifying information or case descriptions for this individual.**

We appreciate the comment and have replaced these images with ones that do not contain any identifying information.

**5. Please review your reference list to ensure that it is complete and correct. If you have cited papers that have been retracted, please include the rationale for doing so in the manuscript text, or remove these references and replace them with relevant current references. Any changes to the reference list should be mentioned in the rebuttal letter that accompanies your revised manuscript. If you need to cite a retracted article, indicate the article’s retracted status in the References list and also include a citation and full reference for the retraction notice.**

The reference list has been checked and is complete and correct. At the time of submission Ref no.15 was in press and has since been published. This publication status has been updated accordingly on pg 31 line 537

**Additional Editor Comments:**

**Thank you for your submission. The study presents the protocol development for a challenging intervention. Acknowledging our first reviewer's comments, protocols are a format PLoS One accepts.

With regards to the criteria for publication:
1. The study presents the results of original research.
2. Results reported have not been published elsewhere.
3. Experiments, statistics, and other analyses are performed to a high technical standard and are described in sufficient detail. The manuscript is a protocol, but also describes protocol development.
4. Conclusions are presented in an appropriate fashion and are supported by the data.
5. The article is presented in an intelligible fashion and is written in standard English.
6. The research meets all applicable standards for the ethics of experimentation and research integrity.
7. The article adheres to appropriate reporting guidelines and community standards for data availability. However, it would be worth the authors explicitly referencing a protocol checklist or guideline such as PRISMA-P or SPIRIT (or any relevant extensions), completing the checklist and submitting as an attachment in their resubmission.**

All of these criteria have been met.

With respect to criteria 5, we have made minor changes and fixed any grammar and tense where necessary to make it cleared to the reader. All changes are tracked.

With respect to item 7, we were unable to find any existing checklists relevant to a protocol development arising from a co-design process; however, should you have a recommendation for a specific checklist that aligns with this format, we will be more than happy to complete and submit one.
